# Gender Differences in the Link Between Informal Caregiving and Subjective Well-Being

**DOI:** 10.1093/geroni/igab046.2931

**Published:** 2021-12-17

**Authors:** Aeri Kim, Kyungmi Woo

**Affiliations:** Seoul National University, Seoul, Seoul-t'ukpyolsi, Republic of Korea

## Abstract

Despite existing evidence on the negative association between informal caregiving and caregiver’s well-being, three important knowledge gaps remain. First, the link warrants further scrutiny due to the possibility of individual heterogeneity. Second, less is known about how informal caregiving is related to caregiver’s well-being. Third, there is little consensus in the literature regarding whether caregiver’s gender matters. This study fills these gaps in the literature. Using seven waves of a large-scale, nationally representative longitudinal study of older adults in Korea between 2006 and 2018, this study employed generalized estimating equations models with a lagged dependent variable as well as fixed effects models. Findings from both models revealed that informal caregiving is negatively associated with subjective well-being, and this association is largely driven by female caregivers. To explore potential mechanisms undergirding this association, we examined the mediating roles of a number of health behaviors. We found that engaging in informal caregiving is associated with a reduction in regular exercise. Results from mediation analyses, however, suggested that regular exercise explains only a moderate amount of the observed association (12% for health-related life satisfaction and 8% for self-rated health). While informal caregiving is obviously a rewarding role, it poses a serious threat to caregiver’s well-being. Findings of this study on gender differences in the well-being consequences of informal caregiving lend support to taking a gender-conscious approach in programs aiming to improve the well-being of informal caregivers.

